# Headache/migraine-related stigma, quality of life, disability, and most bothersome symptom in adults with current versus previous high-frequency headache/migraine and medication overuse: results of the Migraine Report Card survey

**DOI:** 10.1186/s12883-024-03732-x

**Published:** 2024-07-04

**Authors:** Dawn C. Buse, Roger Cady, Amaal J. Starling, Meghan Buzby, Charlie Spinale, Kathy Steinberg, Kevin Lenaburg, Steven Kymes

**Affiliations:** 1 Department of Neurology, Albert Einstein College of Medicine, 1300 Morris Park Avenue, Van Etten 3C12, Bronx, NY 10461 USA; 2 RK Consults, Ozark, MO USA; 3 Missouri State University, Springfield, MO USA; 4 Axon Therapeutics, San Diego, CA USA; 5 Mayo Clinic Arizona, Scottsdale, AZ USA; 6 Coalition for Headache and Migraine Patients (CHAMP), San Rafael, CA USA; 7 The Harris Poll, New York, NY USA; 8 Clusterbusters, Inc, Lombard, IL USA; 9 Lundbeck LLC, Deerfield, IL USA

**Keywords:** Chronic migraine, Patient perspective, Disease stigma, Medication overuse, High-frequency migraine, Harris Poll, Migraine Report Card Survey

## Abstract

**Background:**

High-frequency headache/migraine (HFM) and overuse of acute medication (medication overuse [MO]) are associated with increased disability and impact. Experiencing both HFM and MO can potentially compound impacts, including stigma; however, evidence of this is limited. The objective of this report was to evaluate self-reported stigma, health-related quality of life (HRQoL), disability, and migraine symptomology in US adults with HFM + MO from the Harris Poll Migraine Report Card survey.

**Methods:**

US adults (≥ 18 yrs., no upper age limit) who screened positive for migraine per the ID Migraine™ screener completed an online survey. Participants were classified into “current HFM + MO” (≥ 8 days/month with headache/migraine and ≥ 10 days/month of acute medication use over last few months) or “previous HFM + MO” (previously experienced HFM + MO, headaches now occur ≤ 7 days/month with ≤ 9 days/month of acute medication use). Stigma, HRQoL, disability, and most bothersome symptom (MBS) were captured. The validated 8-item Stigma Scale for Chronic Illnesses (SSCI-8) assessed internal and external stigma (scores ≥ 60 are clinically significant). Raw data were weighted to the US adult population. Statistically significant differences were determined by a standard *t*-test of column proportions and means at the 90% (*p* < 0.1) and 95% (*p* < 0.05) confidence levels.

**Results:**

Participants (*N* = 550) were categorized as having current (*n* = 440; mean age 41.1 years; 54% female; 57% White, not Hispanic; 24% Hispanic; 11% Black, not Hispanic) or previous (*n* = 110; mean age 47.2 years; 49% female; 75% White, not Hispanic; 13% Hispanic; 4% Black, not Hispanic) HFM + MO. Compared to those with previous HFM + MO (21%), adults with current HFM + MO were more likely to experience clinically significant levels of stigma (47%). Men with current HFM + MO (52% compared to men with previous HFM + MO [25%] and women with current [41%] or previous [18%] HFM + MO), non-Hispanic Black (51% compared to White, not Hispanic [45%] and Hispanic [48%] current HFM + MO groups and White, not Hispanic previous HFM + MO [12%]), current HFM + MO aged 18–49 years (50% compared to those with current HFM + MO aged ≥ 50 years [33%] and those with previous HFM + MO aged 18–49 [34%] and ≥ 50 years [4%]), and employed respondents (53% current and 29% previous compared to those not employed [32% current and 12% previous]) reported higher rates of clinically significant stigma. Those with current HFM + MO were more likely to have worse HRQoL and disability due to headache/migraine. Respondents aged ≥ 50 years with current HFM + MO were more likely than respondents aged 18–49 years with current HFM + MO to indicate that their overall quality of life (66% vs. 52%) and their ability to participate in hobbies/activities they enjoy were negatively impacted by headache/migraine (61% vs. 49%). Pain-related symptoms were identified as the MBS.

**Conclusions:**

Together these data suggest that current and previous HFM + MO can be associated with undesirable outcomes, including stigma and reduced HRQoL, which were greatest among people with current HFM + MO, but still considerable for people with previous HFM + MO.

**Graphical abstract:**

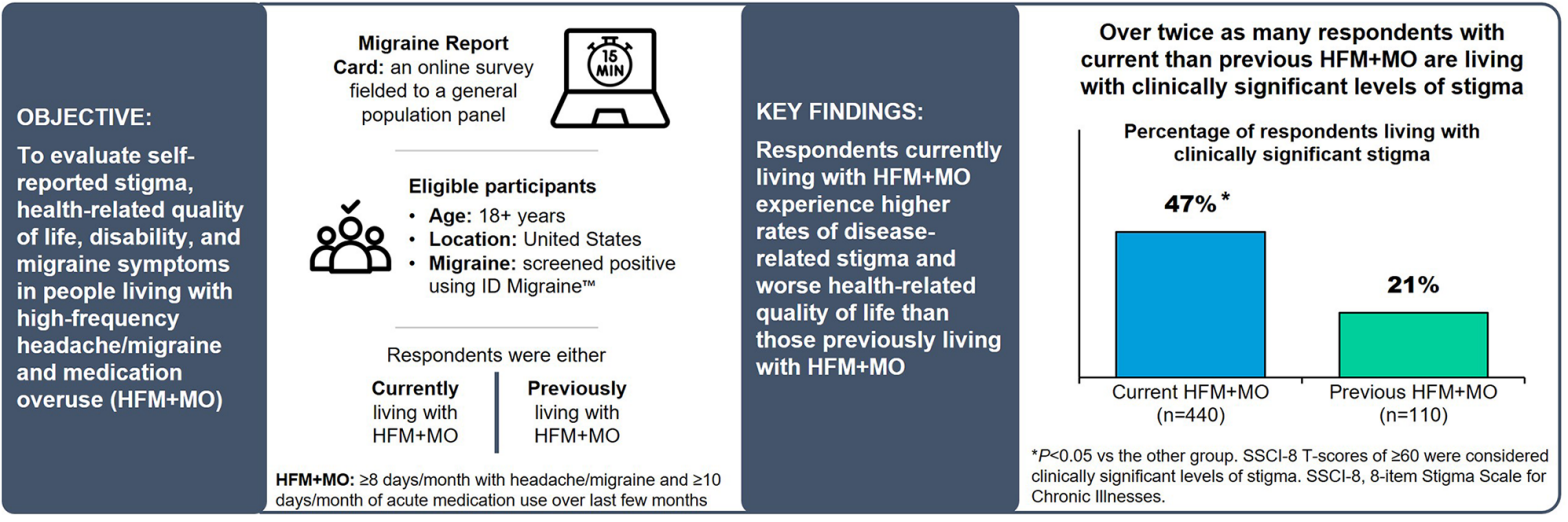

**Supplementary Information:**

The online version contains supplementary material available at 10.1186/s12883-024-03732-x.

## Background

Migraine, the second-leading contributor to years lived with disability, can negatively impact almost all aspects of life, including employment, education, and family and social life [1–3]. Higher-frequency migraine is associated with greater disability and impact, more comorbidities, higher indirect/direct healthcare costs, worse quality of life, and more stigma when compared to those with lower-frequency migraine [4–9]. Moreover, a higher frequency of headache/migraine was found to be associated with less employment and more work productivity impairment when compared to those with a lower frequency of headache/migraine [5, 6].

People using high levels of acute medication to treat migraine attacks or headaches may meet criteria for medication overuse (MO). MO is defined in the International Classification of Headache Disorders-3 (ICHD-3) criteria as either ≥ 10 or ≥ 15 days per month based upon the acute agent(s) used [10–12]. MO is common and associated with many negative outcomes, including risk of progression from episodic migraine to chronic migraine [13].

Stigma refers to negative attitudes and beliefs about a particular group of individuals who share certain characteristics or conditions that deviate from societal norms [14, 15]. Stigma is associated with many medical and psychological diseases, including migraine [16–18]. In headache/migraine, enacted or external stigma refers to negative attitudes and beliefs about people with migraine by others. These attitudes and beliefs may elicit prejudice, discrimination, and/or loss of access to social, economic, and political power [19]. Internalized stigma refers to negative thoughts, feelings, and beliefs that people living with a disease or condition may have about themselves. Internalized stigma can be associated with feelings of shame, guilt, depression, anxiety, and low self-worth. Other types of stigma include structural stigma, medical stigma, and public stigma, among others [20]. There is an emerging body of research showing that migraine-related stigma is common in both population- and clinic-based samples. An analysis of 59,004 respondents to the United States population-based OVERCOME study of people with migraine found that approximately one-third (31.7%) experienced migraine-related stigma often or very often and that higher stigma was associated with lower rates of seeking care [21].

Migraine symptoms can vary across patients and from attack to attack. The concept of “most bothersome symptom” (MBS) attempts to individualize the symptom experience. Clinical trials of acute migraine treatment evaluate an MBS using an investigator-led questionnaire about the incidence of 3 common symptoms. In more recent studies, a patient-identified MBS measurement has been used [22, 23]. In the Migraine Report Card survey, we offered respondents an expanded list of symptoms from which to identify the most bothersome, including headache pain, photophobia, phonophobia, nausea, and cognitive impacts, among others.

Herein, we report findings from a US population-based survey, The Harris Poll Migraine Report Card (“Migraine Report Card”) [24]. The objective of Migraine Report Card was to compare self-reported experiences in the migraine journey of adults with high-frequency headache/migraine and medication overuse (HFM + MO) to those who previously experienced HFM + MO. In this current study we compare the experiences of these two groups using the 8-item Stigma Scale for Chronic Illnesses (SSCI-8) [25] as well as items assessing HRQoL impacts, disability, and MBS. Further, we evaluate stigma experiences and HRQoL impacts within sociodemographic subgroups (i.e., gender, race/ethnicity, age, and employment status) to examine differential effects.

## Methods

### Survey design

Migraine Report Card was an observational, national, cross-sectional online survey administered by The Harris Poll and available to a US general population panel from December 9, 2021, to January 10, 2022. The survey took an estimated 15 min to complete and consisted of closed-ended questions. Respondents were recruited from online market research panels made up of members who agreed to participate in this type of research. Survey respondents provided informed electronic consent prior to screening and were asked to read The Harris Poll privacy policy before agreeing to continue, which included consenting to the results of the survey being published. Only those who selected “I agree to continue” moved on to the screening section. This survey was not intended to provide clinical data for treatment decisions and was not conducted as a clinical trial; therefore, Institutional Review Board approval was not sought nor required. Survey respondents were compensated for their time/participation with loyalty points toward panel membership.

### Participants and selection criteria

**Fig. 1:**
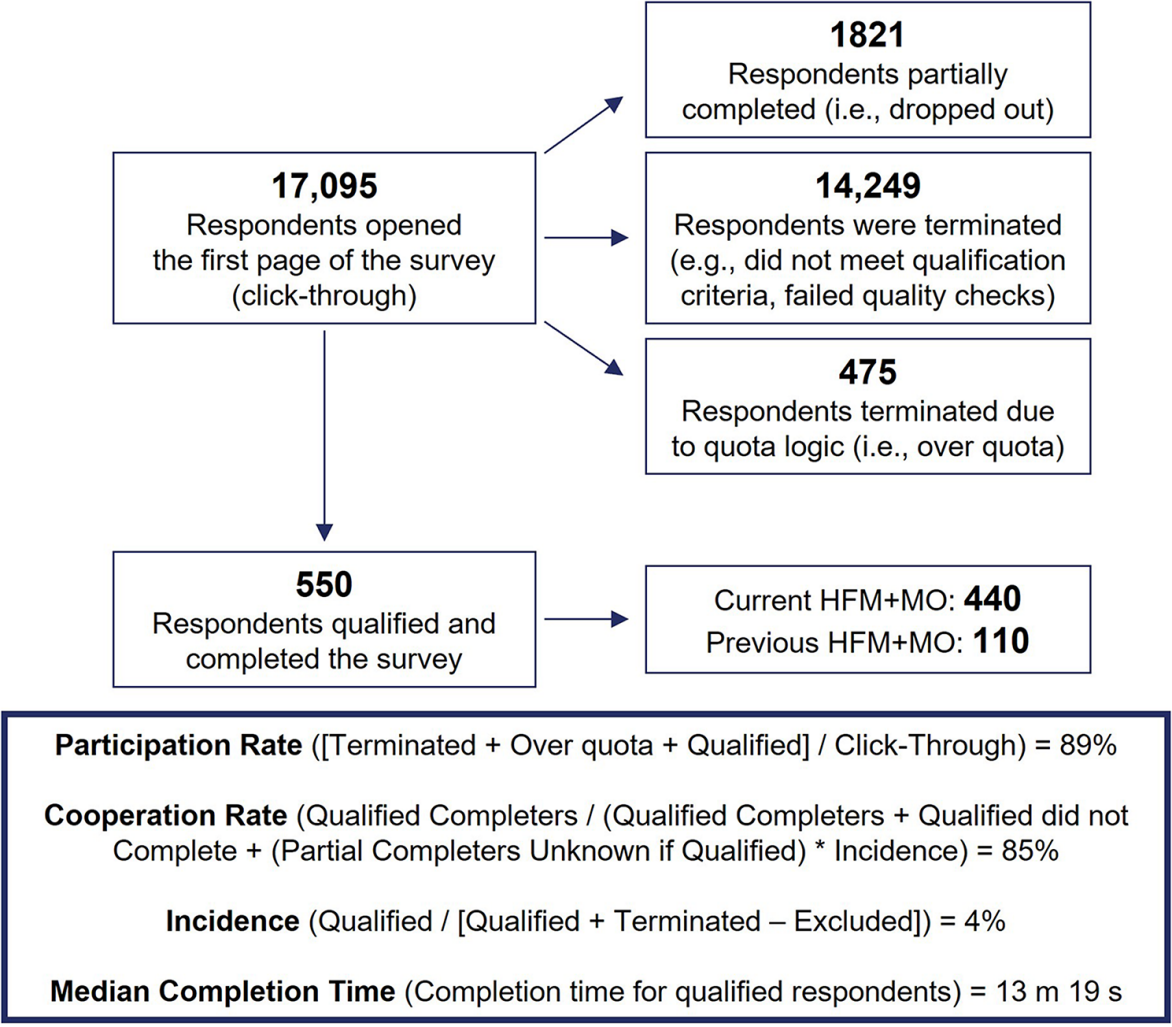
Respondent flow diagram. HFM + MO, high-frequency headache/migraine with medication overuse

To participate in Migraine Report Card, respondents had to be ≥18 years of age (there was no upper age limit) and live in the United States. The Harris Poll recruits participants for its research through a vetted network of trusted sample partners (e.g., both consumer and healthcare provider panels) who assist in recruiting survey participants as needed for each project. Once recruited, interested participants complete a screening questionnaire. Screening questions classified respondents as either “current HFM + MO” or “previous HFM + MO.” Moreover, eligible survey respondents included those who screened positive for migraine based on self-reported ID Migraine™ responses, a validated 3-item screener that identifies individuals very likely to have migraine if they answer “yes” to 2 of the 3 items [26]. The items ask whether headache has limited activities for ≥ 1 day within the past few months, whether nausea is experienced during headache, and whether there is light sensitivity during headache. For “current HFM + MO,” participants self-reported ≥ 8 days/parts of day with headache or migraine per month and ≥ 10 days per month of any acute headache medication use in the last few months. For “previous HFM + MO,” participants self-reported a historical frequency of ≥ 8 days or parts of day with headache or migraine per month, any acute headache medication use ≥ 10 days per month when their headache pattern was at its worst, and now had ≤ 7 days or parts of days with migraine per month in the last few months and ≤ 9 days per month of any acute headache medication use (Fig. 1). In this analysis, HFM + MO is not synonymous with high-frequency episodic migraine as defined in other studies as 10–14 headache days/month or 8–14 headache days/month, chronic migraine, or medication-overuse headache, although respondents could be classified in one or more of those groups. Race and ethnicity were assessed separately; ethnicity was assessed by selecting yes or no to “Are you of Hispanic, Latino, or Spanish origin?” and race was assessed as a “select all that apply” question. For this analysis, race and ethnicity were combined into the following groups: Non-Hispanic White, Non-Hispanic Black, and Hispanic.

### Survey assessments

The Migraine Report Card survey (previously published) [24] included screening questions to assess age, geographic location, headache/migraine history/characteristics, and over-the-counter and/or prescription medication types and quantities. Treatments were those specific to headache/migraine care. Screening also captured demographics (gender, race, and ethnicity), employment status (employed [full-time, part-time], not employed [looking for work, not looking for work, unable to work due to disability/illness], retired, student, stay-at-home spouse/partner), healthcare insurance status, overall health ratings, and comorbidities. Respondents were told to assume that days with “headache” also refer to days with migraine and/or other types of headache. Screening classified participants as current HFM + MO, previous HFM + MO, or neither, in which case the survey was terminated.

Stigma was assessed using the SSCI-8 questionnaire [25]. This questionnaire is composed of 8 questions that assess the amount of internal and perceived external stigma a patient may experience. To each of the 8 items assessed, the following raw scoring was used: 1 = never, 2 = rarely, 3 = sometimes, 4 = often, and 5 = always to give a raw summed score range of 8–40. Raw SSCI-8 scores were then converted to item response theory (IRT)-based T-scores per Molina et al. [25]. SSCI-8 T-scores ≥ 60 were considered clinically significant levels of stigma per Seng et al. [4].

Migraine-related disease burden, including the degree of impact over time, ability to perform everyday activities, most concerning aspects of the disease, disability, and quality of life, were assessed via various questions written for this study. The full survey is available as a supplement in the primary publication [24]. MBS was assessed by asking participants, “Which of the following headache-associated symptoms, if any, do you find to be the most bothersome, other than headache or head pain? Please select one.” There were 24 options to select from, divided by migraine phases: headache (throbbing / pulsation pain, pain, nausea / vomiting, eye pain, neck pain, pain exacerbation with activity, sensory disturbance [e.g., tingling in hands or face, vision changes], anatomical [bodily] pain); prodrome (sensitivity to light, pressure / tightness, sensitivity to sound, sensitivity to smell, sleep disturbance, mood changes, speech difficulty); aura (dizziness, visual impact, aura [e.g., flashing lights, intense head pain, zigzag lines], allodynia [e.g., skin sensitivity when wearing a ponytail, shaving face hurts]); and postdrome (cognitive disruption [e.g., memory problems, difficulty concentrating, feeling fuzzy headed], fatigue, and inactivity). Additionally, participants could choose from “other” or “none.”

### Data collection, weighting, and analysis

The full details on data collection, weighting, and analysis have been previously published [24]. In brief, for this study no formal power calculations were conducted a priori. The sample size was determined based on the want to balance and compare the current and previous arms and what was feasible with the online panel. To ensure statistical comparisons between the two groups could be performed, the quota was set at *n* = 400 (current HFM + MO) and *n* = 100 (previous HFM + MO). These values are also considered to be representative of their respective subgroups in the overall population. The raw data were weighted using the Random Iterative Method (RIM) to align them with their actual proportions in the US population (where necessary). These proportions were identified from benchmarks from the March 2021 US Census Bureau’s Current Population Survey Annual Socioeconomic Supplement by age (18+), education, gender, race, Hispanic ethnicity, US Census region, household income, household size, and marital status [27]. Propensity score weighting was used to adjust for respondents’ propensity to be online.

Specified variables were weighted simultaneously via RIM weighting, but not in combination with one another. RIM weighting provided each respondent with a single weight value, which was capped based on standard parameters by sample size, to limit any extreme weighting or outliers. In this report, unweighted sample sizes are presented; however, percentage values were calculated using weighted data. Statistically significant differences between the current and previous groups were determined by a standard, two-tailed *t*-test of column proportions and means at the 90% (*p* < 0.1) and 95% (*p* < 0.05) confidence levels. Statistical tests were only performed when the sample size was ≥ 30. Sample data are accurate to within + 5.3% points using a 95% confidence level. This credible interval was wider among subsets of the surveyed population of interest. Data were analyzed using IBM® Quantum, version 5.8 (IBM Corporation., Armonk, NY, United States).

In this survey there were no missing data because respondents were required to answer each question (and any subquestions) before moving on to the next question. Respondents were, however, able to decline to answer a response, by selecting “*prefer not to answer*” if it may have been of a sensitive nature. This overall percentage was relatively low across different questions.

## Results

### Survey population and demographics

Of 17,095 individuals screened, a total of 550 US adults were eligible for inclusion and were categorized into either the current HFM + MO group (*n* = 440) or the previous HFM + MO group (*n* = 110) (Fig. 1). Demographics for each group are reported in Table 1. Representation of males and females was balanced in both groups, with a larger representation of males in this survey when compared to other headache/migraine surveys and studies. Over 93% of participants had insurance coverage at the time of the survey. The average number of headache days in the past few months was 15.2 for the current HFM + MO group and 4.2 for the previous group (Supplemental Table 1). Similarly, the average number of days with acute medication usage was 17.4 for the current group and 4.1 for the previous group. Respondents with current HFM + MO were more likely to be employed (66% vs. 54%, *p* < 0.1), specifically employed full-time (56% vs. 42%, *p* < 0.05), than respondents with previous HFM + MO.

**Table 1:** Demographics and clinical characteristics

	Current HFM + MO (*n* = 440) (A)	Previous HFM + MO (*n* = 110) (B)
**Age, mean (SD)**	41.1 (12.9)	47.2 (17.1)^A^
**Gender, %**		
Female	54	49
Male	44	49
Non-binary/Gender non-conforming	1	2
Transgender	1	0
**Race/ethnicity, %** ^*^		
White, not Hispanic	57	75^A^
Hispanic	24^B^	13
Black or African American, not Hispanic	11	4
Asian	2	3
Native American or Alaskan	0	2^A^
More than one race	5	3
**Age at first diagnosis, mean (SD)**	23.9 (11.3)	25.0 (11.1)
**Length of time since diagnosis, mean years (SD)**	17.6 (12.0)	21.9 (15.3)^A^
**Monthly headache days in past few months, mean (SD)**	15.2 (5.8)^B^	4.2 (2.1)
**Monthly acute medication usage, mean (SD) days**	17.4 (6.4)^B^	4.1 (2.3)
**Highest education level completed, %**		
Less than high school	9	4
High school to less than 4-year college degree	57	59
4-year college degree or more	33	37
**Has health insurance, %**		
Yes	93	95
No	7	5
**Employed, %**		
**Yes**	66%^b^	54%
Employed full time	56%^B^	42%
Employed part time	5%	5%
Self-employed full time	3%	3%
Self-employed part time	1%	3%
**No**	34%	46%^a^
Not employed, but looking for work	8%^b^	2%
Not employed and not looking for work	< 1%	2%^A^
Not employed, unable to work due to a disability or illness	10%	7%
Retired	7%	23%^A^
Student	2%	7%^a^
Stay-at-home spouse or partner	6%	5%
**Total yearly household income, %**		
Less than $15,000	7	8
$15,000 to $24,999	8	7
$25,000 to $34,999	9	5
$35,000 to $49,999	9	7
$50,000 to $74,999	19	16
$75,000 to $99,999	13	16
$100,000 or more	34	37

^a/b^Lowercase letters indicate significantly higher than the corresponding group (labeled in the header as A or B) at the 90% confidence level (*p* < 0.1). ^A/B^Uppercase letters indicate significantly higher than the corresponding group (labeled in the header as A or B) at the 95% confidence level (*p* < 0.05)

Percentages may not add up to 100% due to rounding. HFM + MO, high-frequency headache/migraine with medication overuse; SD, standard deviation

^*^Participants were first asked “Are you of Hispanic, Latino, or Spanish origin?” with response options of Yes or No. Then, participants were asked “What is your race? Please select all that apply,” for which the following options were presented White, Black or African American, Native American or Alaskan Native, Native Hawaiian or Pacific Islander, South Asian, Chinese, Korean, Japanese, Filipino, Arab/West Asian, Vietnamese, other Asian, and other race. Data were analyzed by separating Hispanic respondents from the race analysis

### Stigma (Fig. 2)

**Fig. 2:**
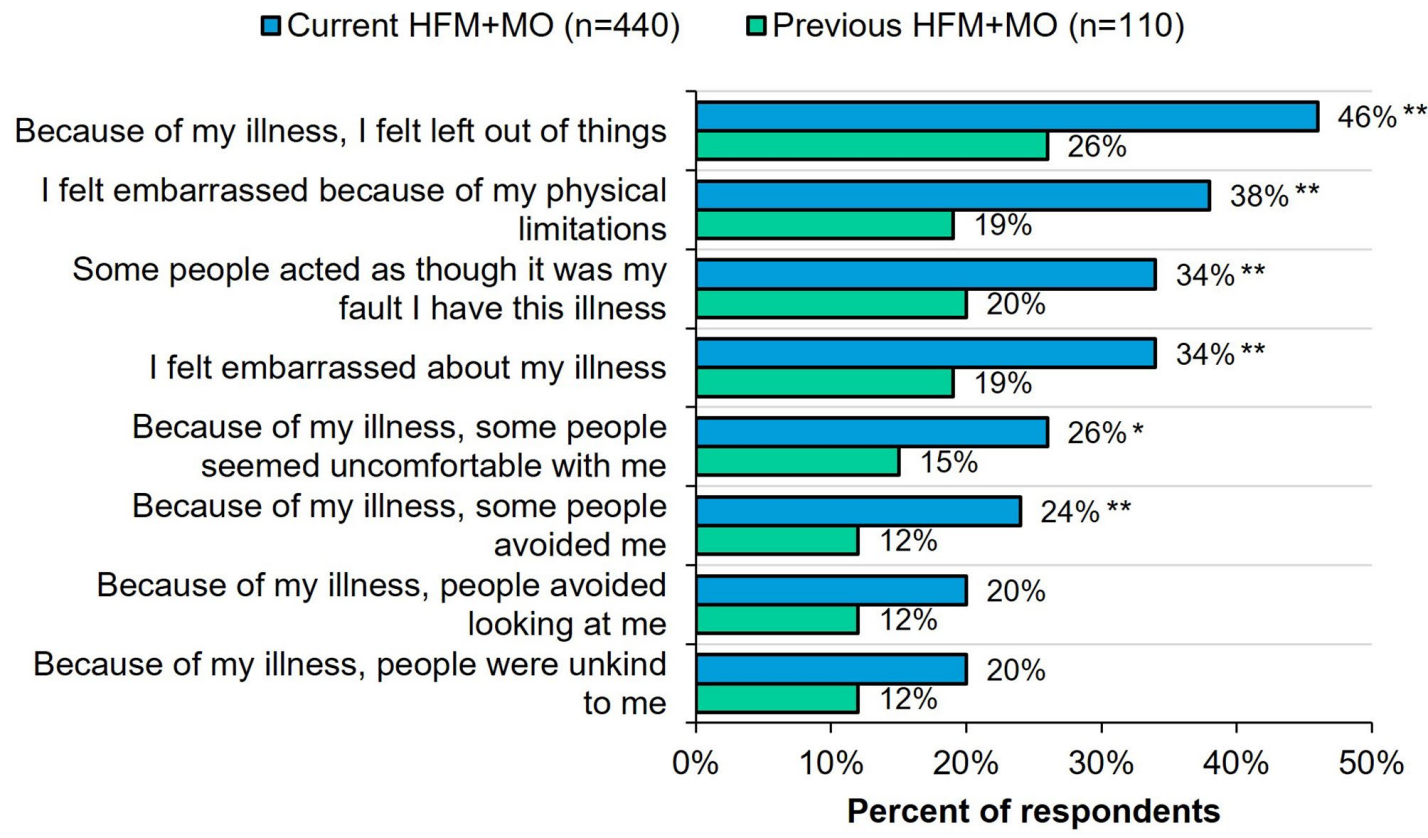
Current vs. previous HFM + MO respondents who selected “always/often” experiencing individual SSCI-8 items. All respondents were asked: “Please indicate how often you experience the following as a result of your headaches.” *Indicates significantly higher than the other group at the 90% confidence level (*p* < 0.1). **Indicates significantly higher than the other group at the 95% confidence level (*p* < 0.05). HFM + MO, high-frequency headache/migraine with medication overuse; SSCI-8, 8-item Stigma Scale for Chronic Illnesses

Compared to those with previous HFM + MO, adults with current HFM + MO were more likely to experience clinically significant levels of stigma (current 47%; previous 21%; *p* < 0.05), yet the rate of stigma among the previous HFM + MO group was still substantial. Participants with current HFM + MO were more likely (*p* < 0.1 or *p* < 0.05) than those with previous HFM + MO to report always/often experiencing 6 of the 8 items on the SSCI-8 due to headache/migraine. Specifically, those with current HFM + MO were more likely to feel left out (*p* < 0.05), feel embarrassed due to physical limitations (*p* < 0.05), feel embarrassed due to headaches in general (*p* < 0.05), believe that some people acted as though headaches were their fault (*p* < 0.05), feel people avoided them due to headache/migraine (*p* < 0.05), and feel that some people seemed uncomfortable with them (*p* < 0.1). There were no differences between current vs. previous HFM + MO regarding the feeling that people avoided looking at them or were unkind to them due to headache/migraine.

### Rates of stigma by gender (table 2)

**Table 2:** Rates of clinically significant stigma by current vs. previous HFM + MO status and gender

	Current HFM + MO		Previous HFM + MO	
	Male (*n* = 181) (A)	Female (*n* = 249) (B)	Male (*n* = 43) (C)	Female (*n* = 63) (D)
**Mean (SD) SSCI-8 T-score**	60.1 (7.9)^B^	57.1 (9.0)	56.6 (12.8)	51.8 (8)
**Respondents with clinically significant stigma (SSCI-8 T-score ≥ 60)**	52%^b^	41%	25%	18%
**Respondents who selected “always/often” experiencing:**				
Because of my illness, I felt left out of things	46%^D^	46%^D^	29%	23%
I felt embarrassed because of my physical limitations	42%^D^	34%^D^	25%	14%
Some people acted as though it was my fault I have this illness	38%^D^	30%^D^	26%	14%
I felt embarrassed about my illness	42%^BD^	27%	22%	16%
Because of my illness, some people seemed uncomfortable with me	29%^D^	25%^D^	22%	8%
Because of my illness, some people avoided me	28%^D^	21%^D^	18%	6%
Because of my illness, people avoided looking at me	29%^BD^	13%^d^	19%	4%
Because of my illness, people were unkind to me	25%^bD^	16%^D^	22%	2%

All respondents were asked: “Please indicate how often you experience the following as a result of your headaches.”

^a/b/c/d^Lowercase letters indicate significantly higher than the corresponding group (labeled in the header as A, B, C, or D) at the 90% confidence level (*p* < 0.1)

^A/B/C/D^Uppercase letters indicate significantly higher than the corresponding group (labeled in the header as A, B, C, or D) at the 95% confidence level (*p* < 0.05)

HFM + MO, high-frequency headache/migraine with medication overuse; SD, standard deviation; SSCI-8, 8-item Stigma Scale for Chronic Illnesses

Men had higher rates of clinically significant headache/migraine-related stigma than women and both men and women with current HFM + MO had higher rates of clinically significant stigma than those with previous HFM + MO (men: 52% vs. 25%; women: 41% vs. 18%). Women with current HFM + MO were more likely (*p* < 0.1 or *p* < 0.05) than women with previous HFM + MO to identify 7 of 8 stigma items on the SSCI-8 as related to their headache/migraine, with no difference in the percentage of women reporting feeling embarrassed about their headache/migraine. For respondents with current HFM + MO, the most commonly identified SSCI-8 item was feeling left out of things (46% for both men and women), followed by feeling embarrassed because of physical limitations (men 42%, women 34%). Specifically, men were more likely than women to feel embarrassed about their headache/migraine (*p* < 0.05) and to feel that people avoided looking at them (*p* < 0.05) and were unkind to them (*p* < 0.1). Small sample sizes precluded comparisons to men with previous HFM + MO (*n* = 43).

### Rates of stigma by race and ethnicity (table 3)

**Table 3:** Rates of stigma by current vs. previous HFM + MO and race/ethnicity

	Current HFM + MO			Previous HFM + MO*
	White, not Hispanic (*n* = 293) (A)	Black, not Hispanic (*n* = 46) (B)	Hispanic (*n* = 75) (C)	White, not Hispanic (*n* = 91) (D)
**Mean (SD) SSCI-8 T-score**	58.0 (8.8)	59.5 (9.0)	58.7 (8.2)	51.2 (8.0)
**Respondents with clinically significant stigma (SSCI-8 T-score ≥ 60)**	45%	51%**	48%	12%
**Respondents who selected “always/often” experiencing:**				
Because of my illness, I felt left out of things	44%^D^	54%^D^	48%^D^	18%
I felt embarrassed because of my physical limitations	35%^D^	51%^aD^	35%^D^	13%
Some people acted as though it was my fault I have this illness	31%^D^	29%^D^	39%^D^	11%
I felt embarrassed about my illness	32%^D^	48%^aD^	35%^D^	11%
Because of my illness, some people seemed uncomfortable with me	25%^D^	34%^D^	29%^D^	10%
Because of my illness, some people avoided me	23%^D^	30%^D^	25%^D^	4%
Because of my illness, people avoided looking at me	19%^D^	22%^D^	21%^D^	6%
Because of my illness, people were unkind to me	17%^D^	30%^aD^	22%^D^	5%

All respondents were asked: “Please indicate how often you experience the following as a result of your headaches.”

^a/b/c/d^Lowercase letters indicate significantly higher than the corresponding group (labeled in the header as A, B, C, or D) at the 90% confidence level (*p* < 0.1)

^A/B/C/D^Uppercase letters indicate significantly higher than the corresponding group (labeled in the header as A, B, C, or D) at the 95% confidence level (*p* < 0.05)

*Due to the base number of respondents being < 30, rates and significance testing were not calculated for Black, not Hispanic, and Hispanic subgroups in the previous HFM + MO group. **Sample size was 55 for this calculation

HFM + MO, high-frequency headache/migraine with medication overuse; SD, standard deviation; SSCI-8, 8-item Stigma Scale for Chronic Illnesses

Non-Hispanic White respondents with current HFM + MO were more likely than their counterparts with previous HFM + MO to have clinically significant stigma (45% vs. 12%) and to identify all 8 stigma items on the SSCI-8. Among those with current HFM + MO, there was a similar percentage (*p* > 0.1) of respondents with clinically significant scores across race/ethnicity groups: non-Hispanic Black, 51%; Hispanic, 48%; non-Hispanic White respondents (45%). The most identified SSCI-8 item was feeling left out of things (non-Hispanic White, 44%; non-Hispanic Black, 54%; Hispanic, 48%) among those with current HFM + MO. Non-Hispanic Black respondents were more likely to feel always/often embarrassed by their headache/migraine (*p* < 0.1) and by the physical limitations associated with headache/migraine than those who are non-Hispanic White with current HFM + MO (*p* < 0.1). In addition, those who are non-Hispanic Black with current HFM + MO were more likely to feel that people were unkind to them (30% vs. 17%, *p* < 0.1). Small sample sizes precluded analysis of Hispanic and non-Hispanic Black respondents in the previous HFM + MO group (*n* < 30).

### Rates of stigma by age (table 4)

**Table 4:** Rates of stigma by current vs. previous HFM + MO and age

	Current HFM + MO		Previous HFM + MO	
	Age 18–49 years (*n* = 327) (A)	Age ≥ 50 years (*n* = 113) (B)	Age 18–49 years (*n* = 56) (C)*	Age ≥ 50 years (*n* = 54) (D)
**Mean (SD) SSCI-8 T-score**	59.2 (8.6)^B^	56.3 (8.2)	57.4 (12.4)	50.1 (6.7)
**Respondents with clinically significant stigma (SSCI-8 T-score ≥ 60)**	50%^B^	33%	34%	4%
**Respondents who selected “always/often” experiencing:**				
Because of my illness, I felt left out of things	48%^D^	41%^D^	34%	16%
I felt embarrassed because of my physical limitations	42%^BD^	26%^D^	28%	9%
Some people acted as though it was my fault I have this illness	41%^BD^	18%^d^	31%	6%
I felt embarrassed about my illness	36%^D^	27%^D^	28%	8%
Because of my illness, some people seemed uncomfortable with me	29%^bD^	18%^d^	22%	6%
Because of my illness, some people avoided me	28%^BD^	14%^D^	21%	1%
Because of my illness, people avoided looking at me	23%^BD^	10%^d^	20%	1%
Because of my illness, people were unkind to me	24%^BD^	8%	22%	0%

All respondents were asked: “Please indicate how often you experience the following as a result of your headaches.”

^a/b/c/d^Lowercase letters indicate significantly higher than the corresponding group (labeled in the header as A, B, C, or D) at the 90% confidence level (*p* < 0.1)

^A/B/C/D^Uppercase letters indicate significantly higher than the corresponding group (labeled in the header as A, B, C, or D) at the 95% confidence level (*p* < 0.05)

*Due to the base number of respondents being < 30, significance testing was not calculated for respondents aged 18–49 years in the previous HFM + MO group

HFM + MO, high-frequency headache/migraine with medication overuse; SD, standard deviation; SSCI-8, 8-item Stigma Scale for Chronic Illnesses

Of those with current HFM + MO, more respondents aged 18–49 years had clinically significant levels of stigma than respondents aged ≥ 50 years (50% vs. 33%]; *p* < 0.05). Respondents aged 18–49 years were also more likely (*p* < 0.1 or *p* < 0.05) than respondents aged ≥ 50 years to select 6 of the 8 stigma items, with no difference between feeling left out of things and feeling embarrassed about their headache/migraine. Similar to the gender-based and race-based analyses, the most common stigma experience for both younger and older respondents with current HFM + MO was feeling left out (younger 48%, older 41%).

Respondents aged ≥ 50 years with current HFM + MO were more likely than respondents aged ≥ 50 years with previous HFM + MO to always/often select 7 of the 8 stigma items, with no difference in feeling people were unkind to them because of their headache/migraine (current 8%, previous 0%). Small sample sizes precluded comparisons to respondents aged 18–49 years in the previous HFM + MO group (*n* < 30).

### Rates of stigma by employment status (table 5)

**Table 5:** Rates of stigma by current vs. previous HFM + MO and employment status

	Current HFM + MO		Previous HFM + MO	
	Employed (*n* = 273) (A)	Not employed (*n* = 167) (B)	Employed (*n* = 47) (C)*	Not employed (*n* = 63) (D)
**Mean (SD) SSCI-8 T-score**	59.7 (8.5)^B^	56.0 (8.2)	56.4 (12.8)	51.6 (7.4)
**Respondents with clinically significant stigma (SSCI-8 T-score ≥ 60)**	53%^B^	32%	29%	12%
**Respondents who selected “always/often” experiencing**				
Because of my illness, I felt left out of things	49%^D^	40%^D^	30%	22%
I felt embarrassed because of my physical limitations	43%^BD^	28%^D^	27%	11%
Some people acted as though it was my fault I have this illness	38%^bD^	28%^D^	28%	10%
I felt embarrassed about my illness	40%^BD^	21%	26%	11%
Because of my illness, some people seemed uncomfortable with me	31%^BD^	17%^d^	23%	5%
Because of my illness, some people avoided me	29%^BD^	14%^D^	21%	2%
Because of my illness, people avoided looking at me	25%^BD^	10%	20%	2%
Because of my illness, people were unkind to me	25%^BD^	10%^d^	22%	1%

All respondents were asked: “Please indicate how often you experience the following as a result of your headaches.”

^a/b/c/d^Lowercase letters indicate significantly higher than the corresponding group (labeled in the header as A, B, C, or D) at the 90% confidence level (*p* < 0.1)

^A/B/C/D^Uppercase letters indicate significantly higher than the corresponding group (labeled in the header as A, B, C, or D) at the 95% confidence level (*p* < 0.05)

*Due to the base number of respondents being < 30, significance testing was not calculated for employed respondents in the previous HFM + MO group

HFM + MO, high-frequency headache/migraine with medication overuse; SD, standard deviation; SSCI-8, 8-item Stigma Scale for Chronic Illnesses

Compared to non-employed respondents with current HFM + MO, more employed respondents with current HFM + MO had clinically significant stigma (53% vs. 32%; *p* < 0.05). Based on employment status, the highest rates of respondents always/often experiencing each of the 8 stigma items were in employed respondents with current HFM + MO, with ≥ 40% reporting always/often feeling left out of things (49%), embarrassed because of physical limitations (43%), and embarrassed about their headache/migraine (40%). Further, employed respondents were more likely (*p* < 0.1 or *p* < 0.05) to select all stigma items, except for feeling left out of things (non-employed 40%, employed 49%). Of non-employed respondents, those with current HFM + MO were more likely (*p* < 0.1 or *p* < 0.05) than those with previous HFM + MO to feel left out of things (*p* < 0.05), feel embarrassed because of physical limitations (*p* < 0.05), and to say that people acted as though it was their fault they have headache/migraine (*p* < 0.05), that people seemed uncomfortable with them (*p* < 0.1), and that people avoided them (*p* < 0.05). Small sample sizes precluded comparisons to employed respondents in the previous HFM + MO group (*n* < 30).

### Headache/migraine-related quality of life, impact, and disability (Fig. 3)

**Fig. 3:**
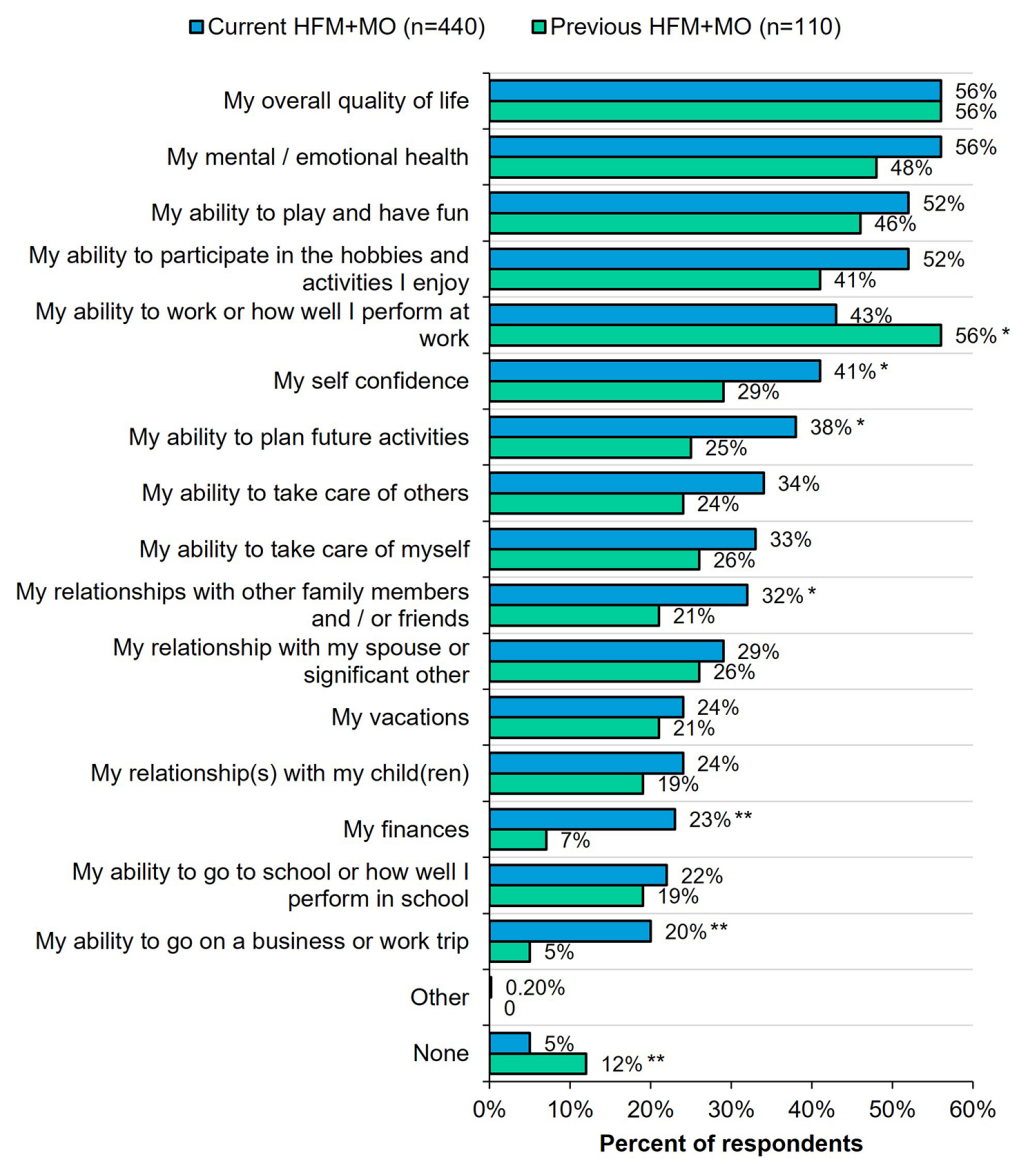
Negative impact of headaches on quality of life and disability by current vs. previous HFM + MO. All respondents were asked: “Do your headaches have a negative impact on any of the following aspects of your life? Please select all that apply.” *Indicates significantly higher than the other group at the 90% confidence level (*p* < 0.1). **Indicates significantly higher than the other group at the 95% confidence level (*p* < 0.05). HFM + MO, high-frequency headache/migraine with medication overuse

When asked if headaches have a negative impact, over half (56%) of respondents in the current and previous HFM + MO groups indicated that headache/migraine negatively impacted their overall HRQoL. In addition, more than half of respondents with current HFM + MO indicated they experienced negative headache/migraine-related impact on mental/emotional health (56%), ability to play and have fun (52%), and ability to participate in enjoyed hobbies/activities (52%). The only impact that a higher percentage of respondents with previous HFM + MO indicated was that headache/migraine negatively impact their ability to work (56% vs. 43%, *p* < 0.1).

### Headache/migraine-related quality of life, impact, and disability examined by gender (Supplemental table 2)

Women with current HFM + MO were more likely than women with previous HFM + MO to indicate that their mental/emotional health (55% vs. 41%, *p* < 0.1), ability to play and have fun (56% vs. 41%, *p* < 0.1), and relationship(s) with other family members and/or friends (35% vs. 17%, *p* < 0.05), self-confidence (35% vs. 18%, *p* < 0.05), and finances (20% vs. 5%, *p* < 0.05) were negatively impacted by headache/migraine. Conversely, women with previous HFM + MO were more likely to indicate no negative impacts due to headache/migraine (17% vs. 5%, *p* < 0.05), although rates were low among both groups. Interestingly, of respondents with current HFM + MO, men were more likely than women to indicate their self-confidence was negatively impacted by headache/migraine (46% vs. 35%, *p* < 0.1). Small sample sizes precluded comparisons to men with previous HFM + MO (*n* < 30).

### Headache/migraine-related quality of life, impact, and disability examined by race and ethnicity (Supplemental table 3)

Of 16 items listed, respondents who were non-Hispanic White with current HFM + MO were more likely to have negative headache/migraine-related impact on 12 quality of life items than respondents who were non-Hispanic White with previous HFM + MO. The top 3 negatively affected aspects of life (due to headache/migraine) for non-Hispanic White respondents with current HFM + MO were mental/emotional health (58%), overall HRQoL (57%), and ability to play and have fun (53%); for non-Hispanic Black respondents with current HFM + MO were ability to participate in enjoyed hobbies/activities (55%), overall quality of life (53%), and mental/emotional health (48%); and for Hispanic respondents with current HFM + MO were overall HRQoL (57%), ability to participate in enjoyed hobbies/activities (54%), and ability to play and have fun (51%). In the current HFM + MO group, more respondents who are non-Hispanic White indicated a negative impact on ability to go to/perform at work (47% vs. 30%, *p* < 0.05) and on relationships with other family members and/or friends (36% vs. 24%, *p* < 0.1) or no negative impacts (7% vs. 0%, *p* < 0.05) than respondents who are Hispanic. Hispanic respondents with current HFM + MO were more likely than non-Hispanic Black respondents with current HFM + MO to indicate a negative impact on vacations (28% vs. 10%, *p* < 0.1). Small sample sizes precluded analysis of Hispanic and non-Hispanic Black respondents in the previous HFM + MO group (*n* < 30).

### Headache/migraine-related quality of life, impact, and disability examined by age (Supplemental table 4)

Respondents aged ≥ 50 years with current HFM + MO were more likely than respondents aged 18–49 years with current HFM + MO to indicate that their overall quality of life (66% vs. 52%, *p* < 0.05), their ability to play/have fun (61% vs. 49%, *p* < 0.1), and their ability to participate in hobbies/activities they enjoy were negatively impacted by headache/migraine (61% vs. 49%, *p* < 0.1). Conversely, respondents aged 18–49 years with current HFM + MO were more likely to indicate that their headache/migraine have a negative impact on their ability to take care of themselves (36% vs. 25%, *p* < 0.1).

Respondents aged ≥ 50 years with current HFM + MO were more likely than respondents aged ≥ 50 years with previous HFM + MO to indicate that their mental/emotional health (61% vs. 34%, *p* < 0.05), their ability to play and have fun (61% vs. 40%, *p* < 0.05), their ability to participate in hobbies and activities they enjoy (61% vs. 38%, *p* < 0.05), their self-confidence (36% vs. 17%, *p* < 0.05), their ability to plan future activities (44% vs. 12%, *p* < 0.05) and take care of others (28% vs. 5%, *p* < 0.05), their relationships with other family members and/or friends (39% vs. 16%, *p* < 0.05), and their relationship with their spouse/significant other (32% vs. 11%, *p* < 0.05) were negatively impacted by headache/migraine. Small sample sizes precluded comparisons to respondents aged 18–49 years in the previous HFM + MO group (*n* < 30).

### Headache/migraine-related quality of life, impact, and disability examined by employment status (Supplemental table 5)

Over 60% of non-employed respondents with current HFM + MO reported a negative impact of headache/migraine on HRQoL (62%) and mental/emotional health (61%). These respondents were more likely than non-employed respondents with previous HFM + MO to have negative headache/migraine-related impact on their ability to play and have fun (56% vs. 30%, *p* < 0.05), to participate in enjoyed hobbies/activities (51% vs. 34%, *p* < 0.1), to plan future activities (37% vs. 20%, *p* < 0.05), to take care of themselves (38% vs. 22%, *p* < 0.1), and to take care of others (35% vs. 19%, *p* < 0.1), as well as negative impact on self-confidence (34% vs. 20%, *p* < 0.1), relationships with other family members and/or friends (34% vs. 20%, *p* < 0.1), and finances (17% vs. 2%, *p* < 0.05). Of those with current HFM + MO, non-employed respondents were more likely to have no negative impacts than employed respondents (8% vs. 3%, *p* < 0.1). Conversely, employed respondents were more likely than non-employed respondents to indicate negative impacts on ability to go to/perform at work (48% vs. 33%, *p* < 0.05), self-confidence (44% vs. 34%, *p* < 0.1), finances (27% vs. 17%, *p* < 0.1), ability to go to/perform at school (25% vs. 14%, *p* < 0.05), and ability to go on a business or work trip (26% vs. 10%, *p* < 0.05). Small sample sizes precluded comparisons to employed respondents in the previous HFM + MO group (*n* < 30).

### Most bothersome symptom (Fig. 4)

**Fig. 4:**
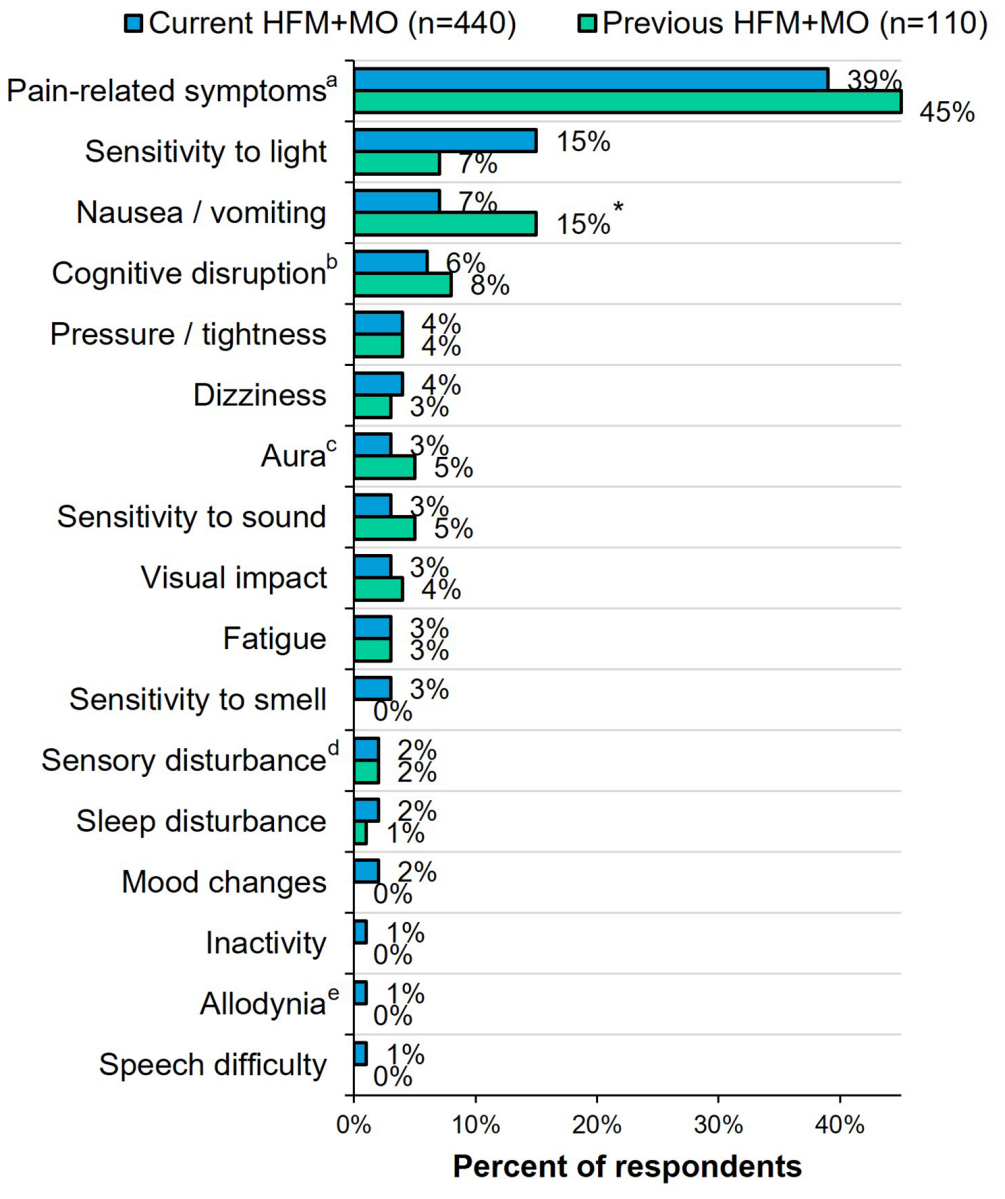
Most bothersome symptom selected by current vs. previous HFM + MO. All respondents were asked: “Which of the following headache-associated symptoms, if any, do you find to be the most bothersome, other than headache or head pain? Please select one.” ^a^Includes, throbbing / pulsation pain, pain, eye pain, neck pain, pain exacerbation with activity, and anatomical (bodily) pain. ^b^Option was “Cognitive disruption (e.g., memory problems, difficulty concentrating, feeling fuzzy headed).” ^c^Option was “Aura (e.g., flashing lights, intense head pain, zigzag lines).” ^d^Option was “Sensory disturbance (e.g., tingling in hands or face, vision changes).” ^e^Option was “Allodynia (e.g., skin sensitivity when wearing a ponytail, shaving face hurts).” *Indicates significantly higher than the other group at the 90% confidence level (*p* < 0.1). HFM + MO, high-frequency headache/migraine with medication overuse

When asked to identify their MBS from a pre-populated list of headache/migraine-associated symptoms, respondents from both groups selected pain-related symptoms which included both headache-qualifying symptoms and pain in other locations (eye and neck pain and pain exacerbation with activity) most often (current 39% vs. previous 45%), followed by sensitivity to light (current 15% vs. previous 7%) and nausea/vomiting (current 7% vs. previous 15%; *p* < 0.1).

## Discussion

In this analysis of data from the Migraine Report Card study, those with current HFM + MO experienced clinically significant stigma at higher rates than adults with previous HFM + MO (21% vs. 47%, respectively), had worse HRQoL, and greater disability due to headache/migraine. In the Migraine Report Card survey primary manuscript, which reported on health status and perception of healthcare among people with current and previous HFM + MO, few distinguishing factors relative to current overall health, mental/emotional health, and healthcare needs between groups were observed [24]. This analysis of the Migraine Report Card study data explored stigma, migraine-related quality of life, migraine-related disability, and MBS between the current and previous HFM + MO groups, as well as by different sociodemographic subgroups (i.e., gender, race/ethnicity, age, and employment status) and found that stigma and reduced HRQoL were greatest among people with current HFM + MO, but still considerable for people with previous HFM + MO.

### Stigma

Respondents in the current HFM + MO group were twice as likely to report the experience of stigma due to headache/migraine than were those with previous HFM + MO, yet it is noteworthy that 1 in 5 respondents with previous HFM + MO still experienced clinically significant levels of stigma. Men with current HFM + MO were more likely to select items such as feeling embarrassed about their illness on the SSCI-8 than were women. Further, employed respondents with current HFM + MO (53% had SSCI-8 T-score ≥ 60) were more likely than those who were unemployed with HFM + MO (32% had SSCI-8 T-score ≥ 60) to select all but one stigma item from the SSCI-8, suggesting that those with HFM + MO who are employed may experience stigma at higher rates.

Among those with current HFM + MO, non-Hispanic Black respondents were more likely than non-Hispanic White respondents to select that headache/migraine made them feel embarrassed and that people were unkind to them on the SSCI-8. The stigma experienced by Black adults may contribute to the lower number of outpatient visits and migraine diagnostic rates among Black populations when compared to Hispanic and White populations found in some studies [28, 29]. Moreover, in an analysis of the CaMEO study, while Black respondents had higher rates of consultation for headache/migraine than White respondents and other racial groups, Black and multiracial respondents had the highest rates of MO. This finding may reflect poorly optimized care or a lack of preventive treatment which could be due to not seeking care because of the stigma experienced [30]. Of note, the differences observed in this study were present despite health insurance status (yes or no) being similar, suggesting that there are factors beyond healthcare access that may be associated with stigma.

Recent population- and clinic-based studies have shown that stigma is common in migraine and is associated with many undesirable outcomes [4, 21, 31]. Stigma is associated with shame, guilt, lower self-esteem, lower self-efficacy, and reduced likelihoods of seeking care and receiving optimized healthcare [20, 32]. The OVERCOME survey assessed how frequently respondents experienced migraine-related stigma via a novel 12-item questionnaire (Migraine-Related Stigma, MiRS) that OVERCOME researchers developed. Approximately 32% of participants (18,708/59,004) experienced at least one type of migraine-related stigma often/very often, and the risk for increased disability (quantified via the Migraine Disability Assessment Scale) was significant for each MiRS group [21]. Similarly to what was observed in the Migraine Report Card, the proportion experiencing migraine-related stigma often/very often increased from 25.5 to 47.5% as monthly headache days increased from < 4 to ≥ 15 [33]. Moreover, in the OVERCOME participant population, migraine-related stigma was associated with poorer quality of life and higher rates of disability, with estimated Migraine Disability Assessment (MIDAS) scores shown to increase as both stigma and monthly headache days increased [33].

A clinic-based study by Seng et al. of 121 adults (≥ 18 years of age) with migraine (meeting criteria for migraine based upon on the American Migraine Study/American Migraine Prevalence and Prevention migraine diagnostic module) recruited from neurology offices in the greater New York City area evaluated stigma using the SSCI-8 as well as several other outcomes. It was shown that almost 20% of respondents had clinically significant levels of stigma [4]. This is similar to the rate we found among people with previous HFM + MO (21%) but lower than the rate of clinically significant stigma we found among people with current HFM + MO (47%). Like the current study, Seng et al. also found that higher SSCI-8 scores were associated with higher headache day frequency, which aligns with the different rates of clinically significant stigma seen in the current and previous HFM + MO groups in the Migraine Report Card survey. Seng et al. opined that higher frequency migraine could provide more opportunities for migraine attacks to interfere with one’s ability to fulfill obligations or engage in social activities. Like OVERCOME, Seng et al. also found that higher SSCI-8 scores were associated with greater migraine-related disability and worse quality of life; however, unlike the current study they did not find an association between SSCI-8 and age, sex, ethnicity, or race [4]. Participants who were employed full time had lower SSCI-8 T-scores (50.5) than people who were not employed full time (54.7). Seng et al. also found that higher stigma scores were associated with greater pain catastrophizing, higher levels of depression and anxiety, and greater ictal cutaneous allodynia [4]. Overall, the results from these studies suggest that stigma is prevalent among people living with migraine.

### Headache/migraine impacts, health-related quality of life, and disability

All respondents in this survey currently or previously had MO. MO can affect up to 50% of people with chronic migraine (≥ 15 days per month for > 3 months) and also a fair percentage of individuals with episodic migraine and is associated with significant health-related burden [34]. In the Chronic Migraine Epidemiology and Outcomes (CaMEO) study, a US-based survey of adults with migraine, those with MO had greater interictal burden when compared to those who did not have MO (65% vs. 32% had moderate-severe interictal burden as measured by the Migraine Interictal Burden Scale). Moreover, those with MO were found to have more severe headache-related disability [11].

More than 50% of respondents with current and previous HFM + MO reported that headache/migraine has a negative impact on their HRQoL, with certain sociodemographic subgroups being more affected than others. In addition, those with a current HFM + MO were more likely to indicate that headache/migraine had a negative impact on self-confidence, planning future activities, relationship with family members/friends, finances, and ability to go on a business/work trip. Similarly to what has been previously shown [6, 9], the differences observed between those with current or previous HFM + MO signify that reducing migraine frequency can rectify many quality-of-life impacts.

Those with previous versus current HFM + MO were just as likely to indicate that headache/migraine negatively impacted their ability to work or how well they perform at work, which could be associated with reduced job performance and loss of productivity [35, 36]. Similarly, data from the CaMEO study found that 32.7% of respondents indicated that headache/migraine negatively affected ≥ 1 career area and 32.1% expressed worry about long-term financial security due to migraine, with women slightly more likely than men to agree migraine affected their career [7]. In addition, non-Hispanic White respondents were more likely to indicate negative impacts on work performance and relationships with other family/friends than Hispanic respondents.

The current analysis of the Migraine Report Card did not attempt to identify a connection between headache/migraine and employment status or occupational disability status. However, the overall rate of unemployment among those in the current HFM + MO group was 34% compared to 46% in the previous HFM + MO group (*p* < 0.10). The rate of “not employed because of disability” was 10% in the current HFM + MO group and 7% in the previous HFM + MO group. These unemployment rates likely do not fully explain this relationship, as respondents may have had to change jobs or modify their workday to accommodate headache/migraine, although this survey did not capture that information. Of note, 7% in the current group and 23% in the previous group were retired. Moreover, there was an age difference between the current and previous groups, with the mean age of the current group being 41.1 years and the mean age of the previous group being 47.2 years, which may account for differences observed in employment status. Further research into how migraine affects HRQoL in different subpopulations is warranted as this can help to shape personalized migraine care.

### Most bothersome symptom

When asked to select their most bothersome migraine symptom from a pre-populated list of potential headache/migraine-associated symptoms, among both the current and previous HFM + MO groups, the most common responses were pain-related symptoms, which included both headache-defining symptoms and pain in other locations. Previous literature has shown that photophobia is the most commonly endorsed symptom, especially when patients are asked to select from the three standard MBS options [23]. However, in a previous study in which patients with chronic migraine self-identified their MBS, patients reported 23 unique symptoms, of which the most common were light sensitivity, nausea/vomiting, and pain with activity [22]. The MBS may be different for individual patients and may change over time. Ensuring that treatments effectively address each person’s symptoms is essential to patient satisfaction and may help improve adherence/persistence and reduce or prevent MO.

### Limitations and strengths

This study has some limitations. All data were collected via self-report in an online survey, meaning that all respondents had to have access to the internet and must have completed a “confirmed” or “double opt-in” process to be included in the study. The nature of a survey is also subject to sources of error including response bias, recall error, social desirability biases, and/or error associated with question wording and response options. No supporting documentation or medical records were collected for verification, and the terms migraine and headache were often used interchangeably throughout survey questions. In this study, there was a potential for recall bias especially among the group of previous HFM + MO respondents. Respondents may not recall migraine frequency, acute medication use frequency, severity, or impact accurately over time. The survey framed many questions as whether respondents had this symptom in the “last few months,” but did not specify a precise number of months; respondents may have responded based on their interpretation of this timeframe (e.g., 2 − 4 months). Race and ethnicity were combined into a single category and participants were divided into three groups for this analysis (Non-Hispanic White, Non-Hispanic Black, and Hispanic). Validated instruments were not used to collect impact, disability of quality-of-life data, but rather individual items asking about a range of potential impacts and important elements of life were assessed. Compared to other US-based surveys, there were fewer respondents (*n* = 550) included in this survey, which may limit data interpretation. Moreover, a significant percentage of respondents who were a part of this survey were insured (≥ 93%), with 34–37% making $100,000 or more per year, and therefore may not be indicative of the overall US migraine population and may also have fewer barriers to healthcare. This participant population was not further subdivided or analyzed by insurance type (e.g., commercial vs. government); therefore, comparisons between participants based upon insurance type could not be determined. We did not assess the full ICHD-3 migraine criteria but rather used the ID Migraine™ screener, which suggests a migraine diagnosis and has a pooled sensitivity estimate of 0.84 (95% confidence interval 0.75–0.90) and specificity of 0.76 (95% confidence interval 0.69–0.83) [37]. In addition, in this survey respondents may have had other headache diagnoses as well. In this survey, the respondent’s healthcare provider referred to the primary provider that treated them for headaches/migraine. Therefore, the type of provider likely varied between respondents, which may have affected each respondent’s care and overall responses.

Strengths of this study include its novel hypothesis and efforts to include a broadly representative sample of people with current or previous MO. It is well established that migraine is associated with substantial disability, impairment, and reduced quality of life [7, 9]. Both clinic-based and population-based studies have shown that migraine-related stigma is common [4, 21, 31]. To our knowledge, these factors have not been analyzed and compared between people with current high-frequency migraine and high acute medication use versus a group that is partially remitted. It is known that MO is associated with many undesirable outcomes, but little work has been done assessing its association with stigma [11, 12]. Moreover, we believe these constructs have not been examined by race, ethnicity, gender, or sociodemographic factors (like employment status).

To our knowledge, this is the first US-population based survey to assess overall migraine-related stigma using a validated tool for chronic illness-related stigma (SSCI-8) and differences based on HFM + MO status, gender, employment status, and race/ethnicity. This study also had a higher representation of typically underrepresented groups in migraine studies: men (44–49%), non-Hispanic Black respondents (4–11%), and Hispanic respondents (13–24%). Moreover, raw data were weighted to the population of US adults age ≥ 18 years by education, age, gender, race, Hispanic ethnicity, US Census region, household income, household size, marital status, and propensity to be online, ensuring that results were projectable to the US population and that the survey’s specific migraine quota groups were representative of their respective subgroups in the overall population.

## Conclusions

In this analysis, individuals with current HFM + MO were more impacted by headache/migraine, but those with previous HFM + MO were also negatively impacted in many ways, including stigma, associated disability, and negative impact on quality of life despite having reported far fewer headache/migraine days per month and utilizing considerably less acute medication. Among those with current HFM + MO, rates of headache/migraine-related stigma were higher in males, adults aged 18–49, non-Hispanic Black, and/or employed adults, and HRQoL was most negatively impacted in men, non-Hispanic White, and respondents ≥ 50 years of age. Together these data suggest that migraine at any frequency can be associated with a broad range of undesirable outcomes and that the impact is greatest among people with current HFM + MO when compared to those with previous HFM + MO. Along with established data on disability associated with migraine and recent work on stigma in migraine, these findings suggest that substantial work is needed to understand and address stigma and disability associated with migraine and MO. Anti-stigma strategies can include public education programs, efforts to speak against injustices caused by stigma, and addressing internalized stigma as a part of the treatment regimen for individual patients [20]. Higher rates of migraine-related stigma among Black respondents seen in this survey demonstrate that healthcare professionals and advocates in the migraine space must continue to increase awareness through community engagement, improved cross-cultural communication, and increased racial and ethnic representation in headache-related studies/research [28, 38, 39]. It is our hope that successful treatment as well as mitigation of migraine disability and stigma may be associated with additional improved outcomes for people living with migraine.

### Electronic supplementary material

Below is the link to the electronic supplementary material.

Supplementary Material 1

Supplementary Material 2

## Data Availability

In accordance with EFPIA’s and PhRMA’s “Principles for Responsible Clinical Trial Data Sharing” guidelines, Lundbeck is committed to responsible sharing of clinical trial data in a manner that is consistent with safeguarding the privacy of patients, respecting the integrity of national regulatory systems, and protecting the intellectual property of the sponsor. The protection of intellectual property ensures continued research and innovation in the pharmaceutical industry. Deidentified data are available to those whose request has been reviewed and approved through an application submitted to https://www.lundbeck.com/global/our-science/clinical-data-sharing.

## Abbreviations

CaMEO: Chronic Migraine Epidemiology and Outcomes Study
HFM: High-frequency migraine
HFM + MO: High-frequency headache/migraine and medication overuse
HRQoL: Health-related quality of life
ICHD-3: International Classification of Headache Disorders 3rd edition
IRT: Item response theory
MBS: Most bothersome symptom
MO: Medication overuse
MiRS: Migraine-Related Stigma
OVERCOME: ObserVational survey of the Epidemiology, tReatment, and Care Of MigrainE
RIM: Random Iterative Method
SSCI-8: 8-Item Stigma Scale for Chronic Illnesses
