# Supplementary figures and images for: Headache/migraine-related stigma, quality of life, disability, and most bothersome symptom in adults with current versus previous high-frequency headache/migraine and medication overuse: results of the Migraine Report Card survey
Source: BMC Neurol. 2024 Jul 4;24:232. doi: 10.1186/s12883-024-03732-x (PMC11223432; doi:10.1186/s12883-024-03732-x)

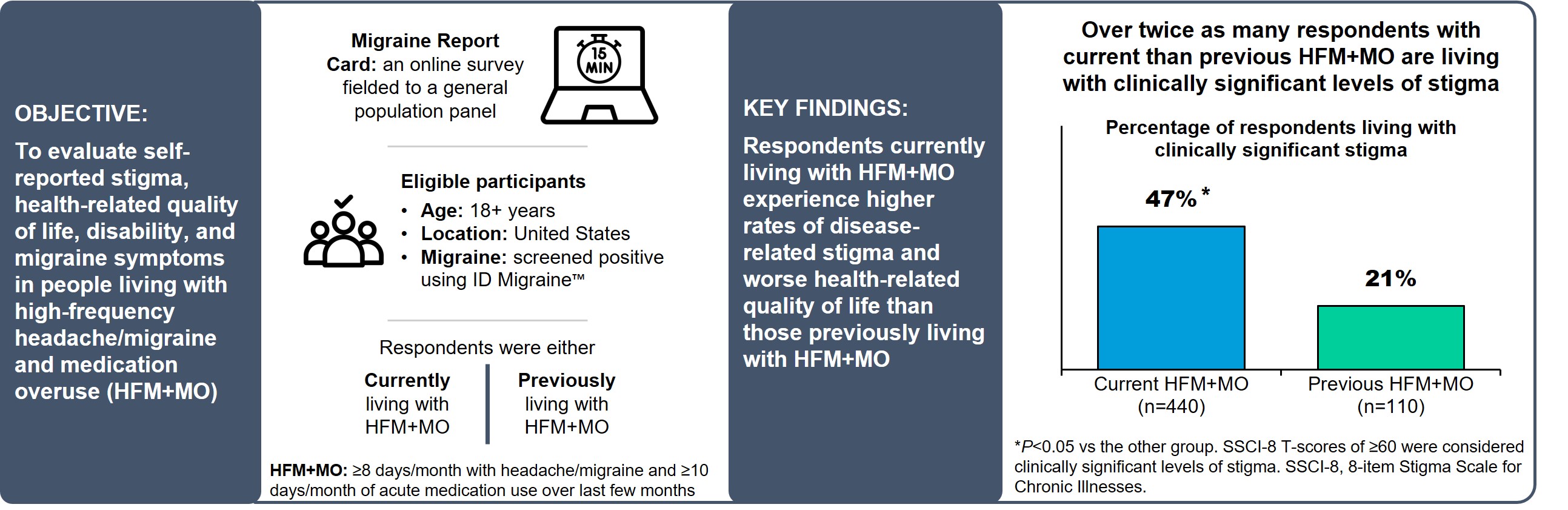

Supplement: Supplementary file 1 — Supplementary Material 1 [file 12883_2024_3732_MOESM1_ESM.jpg]
