# Supplementary material for: Headache/migraine-related stigma, quality of life, disability, and most bothersome symptom in adults with current versus previous high-frequency headache/migraine and medication overuse: results of the Migraine Report Card survey
Source: BMC Neurol. 2024 Jul 4;24:232. doi: 10.1186/s12883-024-03732-x (PMC11223432; doi:10.1186/s12883-024-03732-x)
Supplement: Supplementary file 2 — Supplementary Material 2 [file 12883_2024_3732_MOESM2_ESM.pdf]

Supplemental Materials

Supplemental Table 1. Days per month that respondents experienced a headache

Number of headache days/month reported	**Current HFM+MO (n=440) (A)**	**Previous HFM+MO (n=110) (B)**
**0 days, %**	0%	9%^{A}
**1–9 days, %**	7%	91%^{A}
**10 days, %**	28%^{B}	0%
**11–14 days, %**	14%^{B}	0%
**15 days, %**	16%^{B}	0%
**16–19 days, %**	7%^{B}	0%
**20 days, %**	13%^{B}	0%
**21+ days, %**	15%^{B}	0%
**Mean, days**	15.2^{B}	4.2

All respondents were asked: "Thinking about the last few months, how many days per month have you experienced a headache? Please include ALL days with headache pain of any kind, including migraine, lasting more than 30 minutes."

$^{A/B}$Uppercase letters indicate significantly higher than the corresponding group (labeled in the header as A or B) at the 95% confidence level (p<0.05).

Supplemental Table 2. Negative impact of headache/migraine on quality of life, by current vs. previous HFM+MO and gender

	**Current HFM+MO**		**Previous HFM+MO**	
	**Male (n=181) (A)**	**Female (n=249) (B)**	**Male (n=43) (C)***	**Female (n=63) (D)**
My overall quality of life	56%	55%	58%	53%
My mental / emotional health	55%[d]	55%[d]	55%	41%
My ability to play and have fun	48%	56%[d]	51%	41%
My ability to participate in the hobbies and activities I enjoy	51%	53%	39%	43%
My ability to work or how well I perform at work	45%	42%	69%	42%
My self confidence	46%[bD]	35%[D]	40%	18%
My ability to plan future activities	42%[D]	34%	29%	22%
My ability to take care of others	29%	38%	21%	26%
My ability to take care of myself	32%	34%	27%	27%
My relationships with other family members and / or friends	30%	35%[D]	23%	17%
My relationship with my spouse or significant other	29%	29%	26%	24%
My vacations	28%	21%	23%	19%
My relationship(s) with my child(ren)	22%	27%	18%	20%
My finances	27%[D]	20%[D]	7%	5%
My ability to go to school or how well I perform in school	21%	22%	25%	12%
My ability to go on a business or work trip	23%[D]	19%[D]	3%	6%
Other	1%	0%	0%	1%
None	4%	5%	7%	17%[AB]

All respondents were asked: "Do your headaches have a negative impact on any of the following aspects of your life? Please select all that apply."

[a/b/c/d]Lowercase letters indicate significantly higher than the corresponding group (labeled in the header as A, B, C, or D) at the 90% confidence level ($p<0.1$).

[A/B/C/D]Uppercase letters indicate significantly higher than the corresponding group (labeled in the header as A, B, C, or D) at the 95% confidence level ($p<0.05$).

*Due to the base number of respondents being <30, significance testing was not calculated for males in the previous HFM+MO group.

HFM+MO, high-frequency headache/migraine with medication overuse.

Supplemental Table 3. Negative impact of headache/migraine on quality of life, by current vs. previous HFM+MO and race

	**Current HFM+MO**			**Previous HFM+MO***
	**White, not Hispanic (n=293) (A)**	**Black, not Hispanic (n=46) (B)**	**Hispanic (n=75) (C)**	**White, not Hispanic (n=91) (D)**
My overall quality of life	57%	53%	57%	50%
My mental / emotional health	58%[D]	48%	47%	41%
My ability to play and have fun	53%	39%	51%	44%
My ability to participate in the hobbies and activities I enjoy	49%[d]	55%[d]	54%[d]	36%
My ability to work or how well I perform at work	47%[C]	46%	30%	50%[C]
My self confidence	40%[D]	47%[D]	30%	20%
My ability to plan future activities	37%[D]	32%	39%[D]	17%
My ability to take care of others	32%[D]	25%	35%[D]	12%
My ability to take care of myself	32%[D]	23%	34%[d]	19%
My relationships with other family members and / or friends	36%[cD]	22%	24%	15%
My relationship with my spouse or significant other	32%[BD]	10%	24%	14%
My vacations	23%[d]	10%	28%[bd]	12%
My relationship(s) with my child(ren)	23%[D]	27%	25%[d]	11%
My finances	24%[D]	22%[d]	20%[d]	6%
My ability to go to school or how well I perform in school	17%	27%[d]	24%[d]	10%
My ability to go on a business or work trip	21%[D]	10%	25%[D]	5%
Other	1%	0%	0%	1%
None	7%[C]	1%	0%	15%[aBC]

All respondents were asked: "Do your headaches have a negative impact on any of the following aspects of your life? Please select all that apply."

[a/b/c/d]Lowercase letters indicate significantly higher than the corresponding group (labeled in the header as A, B, C, or D) at the 90% confidence level ($p<0.1$).

[A/B/C/D]Uppercase letters indicate significantly higher than the corresponding group (labeled in the header as A, B, C, or D) at the 95% confidence level ($p<0.05$).

*Due to the base number of respondents being <30, rates and significance testing were not calculated for Black, nor Hispanic and Hispanic subgroups in the previous HFM+MO group.

HFM+MO, high-frequency headache/migraine with medication overuse.

Supplemental Table 4. Negative impact of headache/migraine on quality of life, by current vs. previous HFM+MO and age

	**Current HFM+MO**		**Previous HFM+MO**	
	**Age 18–49 years (n=327) (A)**	**Age ≥50 years (n=113) (B)**	**Age 18–49 years (n=56) (C)***	**Age ≥50 years (n=54) (D)**
My overall quality of life	52%	66%^{A}	58%	54%
My mental / emotional health	54%^{D}	61%^{D}	59%	34%
My ability to play and have fun	49%	61%^{aD}	51%	40%
My ability to participate in the hobbies and activities I enjoy	49%	61%^{aD}	43%	38%
My ability to work or how well I perform at work	44%	42%	66%	43%
My self confidence	42%^{D}	36%^{D}	39%	17%
My ability to plan future activities	36%^{D}	44%^{D}	36%	12%
My ability to take care of others	35%^{D}	28%^{D}	39%	5%
My ability to take care of myself	36%^{bD}	25%	36%	13%
My relationships with other family members and / or friends	30%^{d}	39%^{D}	25%	16%
My relationship with my spouse or significant other	28%^{D}	32%^{D}	38%	11%
My vacations	25%^{D}	21%	29%	9%
My relationship(s) with my child(ren)	25%^{d}	21%	25%	13%
My finances	26%^{D}	17%^{D}	11%	1%
My ability to go to school or how well I perform in school	25%^{BD}	12%	29%	6%
My ability to go on a business or work trip	22%^{D}	15%^{D}	9%	0
Other	0%	0%	1%	0%
None	4%	5%	4%	21%^{AB}

All respondents were asked: "Do your headaches have a negative impact on any of the following aspects of your life? Please select all that apply."

$^{a/b/c/d}$Lowercase letters indicate significantly higher than the corresponding group (labeled in the header as A, B, C, or D) at the 90% confidence level (p<0.1).

$^{A/B/C/D}$Uppercase letters indicate significantly higher than the corresponding group (labeled in the header as A, B, C, or D) at the 95% confidence level (p<0.05).

*Due to the base number of respondents being <30, significance testing was not calculated for respondents aged 18–49 years in the previous HFM+MO group.

HFM+MO, high-frequency headache/migraine with medication overuse.

Supplemental Table 5. Negative impact of headache/migraine on quality of life, by current vs. previous HFM+MO and employment

	**Current HFM+MO**		**Previous HFM+MO**	
	**Employed (n=273) (A)**	**Not employed (n=167) (B)**	**Employed (n=47) (C)***	**Not employed (n=63) (D)**
My overall quality of life	53%	62%	55%	58%
My mental / emotional health	53%	61%	48%	48%
My ability to play and have fun	50%[D]	56%[D]	60%	30%
My ability to participate in the hobbies and activities I enjoy	53%[D]	51%[d]	47%	34%
My ability to work or how well I perform at work	48%[Bd]	33%	75%	34%
My self confidence	44%[bD]	34%[d]	37%	20%
My ability to plan future activities	39%[D]	37%[D]	30%	20%
My ability to take care of others	33%[d]	35%[d]	28%	19%
My ability to take care of myself	30%	38%[d]	30%	22%
My relationships with other family members and / or friends	31%	34%[d]	22%	20%
My relationship with my spouse or significant other	29%	28%	30%	21%
My vacations	26%	21%	23%	18%
My relationship(s) with my child(ren)	25%	22%	20%	19%
My finances	27%[bD]	17%[D]	11%	2%
My ability to go to school or how well I perform in school	25%[BD]	14%	26%	10%
My ability to go on a business or work trip	26%[BD]	10%	6%	4%
Other	0%	0%	1%	0%
None	3%	8%[a]	4%	21%[AB]

All respondents were asked: "Do your headaches have a negative impact on any of the following aspects of your life? Please select all that apply."

[a/b/c/d]Lowercase letters indicate significantly higher than the corresponding group (labeled in the header as A, B, C, or D) at the 90% confidence level (p<0.1).

[A/B/C/D]Uppercase letters indicate significantly higher than the corresponding group (labeled in the header as A, B, C, or D) at the 95% confidence level (p<0.05).

*Due to the base number of respondents being <30, significance testing was not calculated for employed respondents in the previous HFM+MO group.

HFM+MO, high-frequency headache/migraine with medication overuse.
